# Evolution and Expression of the Meprin and TRAF Homology Domain-Containing Gene Family in Solanaceae

**DOI:** 10.3390/ijms24108782

**Published:** 2023-05-15

**Authors:** Yangshuo Dai, Sirui Ma, Yixian Guo, Xue Zhang, Di Liu, Yan Gao, Chendong Zhai, Qinfang Chen, Shi Xiao, Zhenfei Zhang, Lujun Yu

**Affiliations:** 1 State Key Laboratory of Biocontrol, Guangdong Key Laboratory of Plant Resources, School of Life Sciences, Sun Yat-sen University, Guangzhou 510275, China; daiysh@gdppri.com (Y.D.); masr469@163.com (S.M.); guoyx67@mail2.sysu.edu.cn (Y.G.); cherxuer@163.com (X.Z.); liud47@mail2.sysu.edu.cn (D.L.); chenqf3@mail.sysu.edu.cn (Q.C.); xiaoshi3@mail.sysu.edu.cn (S.X.); 2 Guangdong Provincial Key Laboratory of High Technology for Plant Protection, Plant Protection Research Institute, Guangdong Academy of Agricultural Sciences, Guangzhou 510640, China; zikzin2020@163.com (Y.G.); zhaichendongya@163.com (C.Z.)

**Keywords:** MATH, evolution, expression profile, Solanaceae

## Abstract

Meprin and TRAF homology (MATH)-domain-containing proteins are pivotal in modulating plant development and environmental stress responses. To date, members of the MATH gene family have been identified only in a few plant species, including *Arabidopsis thaliana*, *Brassica rapa*, maize, and rice, and the functions of this gene family in other economically important crops, especially the Solanaceae family, remain unclear. The present study identified and analyzed 58 MATH genes from three Solanaceae species, including tomato (*Solanum lycopersicum*), potato (*Solanum tuberosum*), and pepper (*Capsicum annuum*). Phylogenetic analysis and domain organization classified these MATH genes into four groups, consistent with those based on motif organization and gene structure. Synteny analysis found that segmental and tandem duplication might have contributed to MATH gene expansion in the tomato and the potato, respectively. Collinearity analysis revealed high conservation among Solanaceae MATH genes. Further *cis*-regulatory element prediction and gene expression analysis showed that Solanaceae MATH genes play essential roles during development and stress response. These findings provide a theoretical basis for other functional studies on Solanaceae MATH genes.

## 1. Introduction

Tumor necrosis factor receptor (TNFR)-associated factors (TRAFs), initially found to interact with and regulate various TNFRs [1], often function as molecular adaptors or E3 ubiquitin ligases that modulate signal transduction from upstream-associated receptors to their different downstream substrates [2,3]. Typically, TRAFs are characterized by a TRAF domain at the C-terminal region, containing about 180 amino acids that form a fold of seven to eight anti-parallel β-strands that regulate protein processing and interaction [3,4,5]. Meprins, a family of membrane-bound and secreted astacin metalloproteases, have a C-terminal domain that is highly homologous and forms a folding structure similar to the TRAF domain [5,6]. Hence, Meprin and TRAF homology proteins have been termed as MATH-domain-containing proteins.

In eukaryotes, MATH-domain-containing proteins have been associated with other functional domains, such as ubiquitin protease (UBP), pox virus and zinc finger/broad-complex, tramtrack and bric-a-brac (POZ/BTB), filamin and RluA, peptidases, tripartite motif (TRIM), astacin, and a really interesting new gene (RING) and zinc finger [5]. Studies based on phylogenetic analysis and domain organization have classified MATH proteins into eight families: the USP7 family, the MATHd/RluA family, the MATHd-only family, the MATHd/BTB family, MATHd/Filament family, the TRAF family, the TRIM37 family, and the Meprin family [5]. However, the MATHd/RluA family, TRAF family, TRIM37 family, and Meprin family have only been found in animals, not in green plants [5,7]. In *Brassica rapa*, MATH proteins have been grouped into six classes [7], whereas, in *Arabidopsis thaliana* and rice (*Oryza sativa*), they have been mainly clustered into four distinct groups, including MATH-only proteins, MATH-BPM proteins, MATH-UBP proteins, and MATH-PEARLI-4 proteins [8].

Advances in sequencing technology have led to the identification of MATH-encoding genes in Arabidopsis, *Brassica rapa*, maize (*Zea mays*), and rice [7,8,9,10,11]. In addition, MATH-BPM proteins, a specifically expanded subfamily of MATH in Poaceae, have been identified in species such as rice, *Brachypodium distachyon*, maize, and *Sorghum bicolor* [12]. However, the genome-wide comprehensive identification of Solanaceae *MATH* genes has not been reported. Solanaceae is an economically important plant family, of which tomato (*Solanum lycopersicum*), potato (*Solanum tuberosum*), and pepper (*Capsicum annuum*) are three representative species [13]. Among them, the potato has been considered a dominant food crop closely associated with global food security, and the genome sequence of this crop has been completed [14]. Tomato is an important fruit crop and a model species used for studies on fruit development. It is feasible for functional genomic analysis since the high-quality genome sequence has been published [15]. Furthermore, the genome sequence of pepper, an economically important crop and one of the oldest domesticated crops, has been reported by two independent research groups from China and Korea [16,17]. These completed genome assemblies make it possible to identify the *MATH* gene family and investigate their functions in Solanaceae.

Several researchers have reported that MATH proteins play pivotal roles in regulating development, phytohormone signaling, and biotic and abiotic stress responses in plants [8,9]. For instance, two redundant MATH-domain-only proteins in Arabidopsis, TARF1a and TARF1b (also named MUSE13 and MUSE14), have been identified as molecular adaptors associated with E3 ligases that regulate immune response and autophagy via ubiquitination and degradation of different downstream substrates [18,19]. The stability of these two proteins is modulated by upstream regulators through different post-translational modifications to regulate autophagy and immune response [20,21]. Two other MATH-UBP-type proteins in Arabidopsis, AtUBP12 and AtUBP13, function as deubiquitinase to maintain protease activity of distinct substrates to positively or negatively regulate physiological processes associated with leaf development [22,23], root development [24], flowering and circadian rhythms [25], stress and phytohormone responses during bacterial invasion [26], nitrogen deficiency [27,28], and UV-induced and jasmonic acid (JA) signaling [29]. The functions and underlying molecular mechanisms of MATH proteins in Arabidopsis have been well investigated; however, information about this large protein family, including its members and their potential roles in other plant species, is limited.

The present study surveyed the full complement of MATH-protein-encoding genes from genomes of three Solanaceae species, including potato, tomato, and pepper. We further analyzed the phylogeny and domains of the identified members to elucidate the phylogenetic relationship of this gene family in Solanaceae. Subsequently, we examined the segmental and tandem duplications in these *MATH* genes in each genome and the intraspecific collinearity relationships to assess evolutionary relationships and expansion force in Solanaceae. Furthermore, we determined the expression profiles of these genes using previously published transcriptome data from various tissues at different development stages and under stress and phytohormone treatment. The study’s findings will provide a reference for further research on the functions of *MATH* genes in Solanaceae during development and stress response.

## 2. Results

### 2.1. Identification of MATH Genes in Solanaceae Genomes

We first performed a combination of Basic Local Alignment Tool for Protein (BLASTP) and HHMER searches using the Pfam MATH domain (PF00917) to query the genomes of eight plant species, including two green algae (*Ostreococcus lucimarinus* and *Chlamydomonas reinhardtii*), one moss (*Physcomitrium patens*), one spikemoss (*Selaginella moellendorffii*), one eudicot (*Arabidopsis thaliana*), and three representative Solanaceae species (tomato, potato, and pepper) (Table 1). The candidate members were screened for the presence of the MATH domain using the Simple Modular Architecture Research Tool (SMART) and the Conserved Domains Database (CDD). A total of 161 sequences were retained as putative MATH genes (Table 1 and Table S1), including 11 genes in both green algae.

The candidate MATH genes included 58 members from the three Solanaceae genomes: 18 in tomato (*SlMATH*), 19 in potato (*StMATH*), and 21 in pepper (*CaMATH*) (Table 1 and Table S2). According to their locations on the chromosomes, these Solanaceae *MATH* genes were designated as *SlMATH01*–*SlMATH18*, *StMATH01*–*StMATH19*, and *CaMATH01*–*CaMATH21* in tomato, potato, and pepper (Table S2). Further characteristic analysis revealed that the length of Solanaceae MATH proteins ranged from 142 amino acids (aa; CaMATH13) to 1691 aa (SlMATH08), molecular weight (Mw) ranged from 16.5 to 189.5 kDa (Table S2), and the theoretical isoelectric point (pI) values ranged from 5.22 (CaMATH18) to 9.77 (CaMATH01) (Table S2). We also found that these Solanaceae MATH proteins were mostly located in the nucleus and/or cell membrane, except for CaMATH05/CaMATH10/CaMATH13 found in the chloroplast (Table S2).

### 2.2. Phylogenetic Relationship, Conserved Motifs/Domains, and Exon–Intron Organization of Solanaceae MATHs

**Figure 1:**
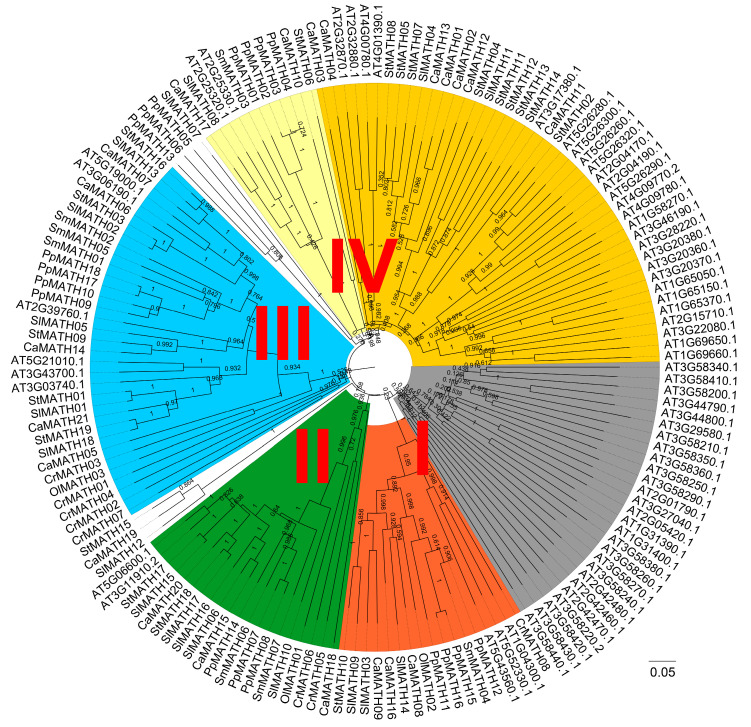
Unrooted phylogenetic tree of plant MATH proteins. Phylogenetic relationships were derived using the maximum likelihood method and JTT matrix-based model in MEGA v11 software. The MATH catalytic domains of eight selected plant species, including two green algae (*Ostreococcus lucimarinus* and *Chlamydomonas reinhardtii*), one moss (*Physcomitrium patens*), one spikemoss (*Selaginella moellendorffii*), one eudicot (*Arabidopsis thaliana*), and three Solanaceae species (*Solanum lycopersicum*, *Solanum tuberosum*, and *Capsicum annuum*), were used in this analysis. The phylogenetic tree was drawn and optimized with the FigTree v1.4.4 software; different colors indicate the six subclades of four distinct groups (groups I–IV) of the MATH proteins. There are seven MATH proteins in the colorless segment, with three members (StMATH15, CaMATH19, and SlMATH12) between groups II and III, and four members (SlMATH07, PpMATH05, PpMATH06, and PpMATH13) between groups III and IV, respectively.

To elucidate the evolutionary relationships of the MATH protein family in plants, we constructed a maximum likelihood (ML) phylogenetic tree using the MATH domain sequences of 161 identified MATH proteins (Figure 1). The phylogenetic tree and domain analysis (Table S1) classified these MATH proteins into four groups (groups I–IV; Figure 1). A total of 27 Arabidopsis MATH proteins were clustered into a subclade of group I, whereas 17 members, including four from moss; three each from Arabidopsis, pepper, and tomato; and one each from *C*. *reinhardtii*, *O*. *lucimarinus*, potato, and spikemoss, were clustered into the other subclade of group I (Figure 1). All these proteins in group I were MATH-only proteins, most containing a single-MATH domain, except PpMATH11 and At3g58420, with two-MATH domains (Table S1). Group II had 20 MATH members from the eight plant species, including 19 MATH-USP7 proteins and CrMATH05, a MATH-BTB protein (Figure 1 and Table S1). Similarly, group III consisted of 34 MATH members from all eight species, including 32 MATH-BTB proteins and two MATH-only proteins (CrMATH01 and CrMATH04) with a single-MATH domain (Figure 1 and Table S1). Another 56 MATH-only proteins from plant species excluding *C*. *reinhardtii* and *O*. *lucimarinus* belonged to group IV, with two subclades. One subclade consisted of ten four-MATH-domain proteins and one single-MATH-domain protein, and the other had 45 MATH members, including 37 two-MATH-domain proteins, six single-MATH-domain proteins (At1G65050/At1G65370/At1G69660, CaMATH02/CaMATH13, and SlMATH11), and two three-MATH-domain proteins (StMATH04 and StMATH12) (Figure 1 and Table S1). One MATH member of *O*. *lucimarinus* existed in the three groups (groups I–III) but not in group IV (Figure 1), suggesting that single-MATH proteins, MATH-USP7 proteins, and MATH-BTB proteins are the three types conserved in plants.

**Figure 2:**
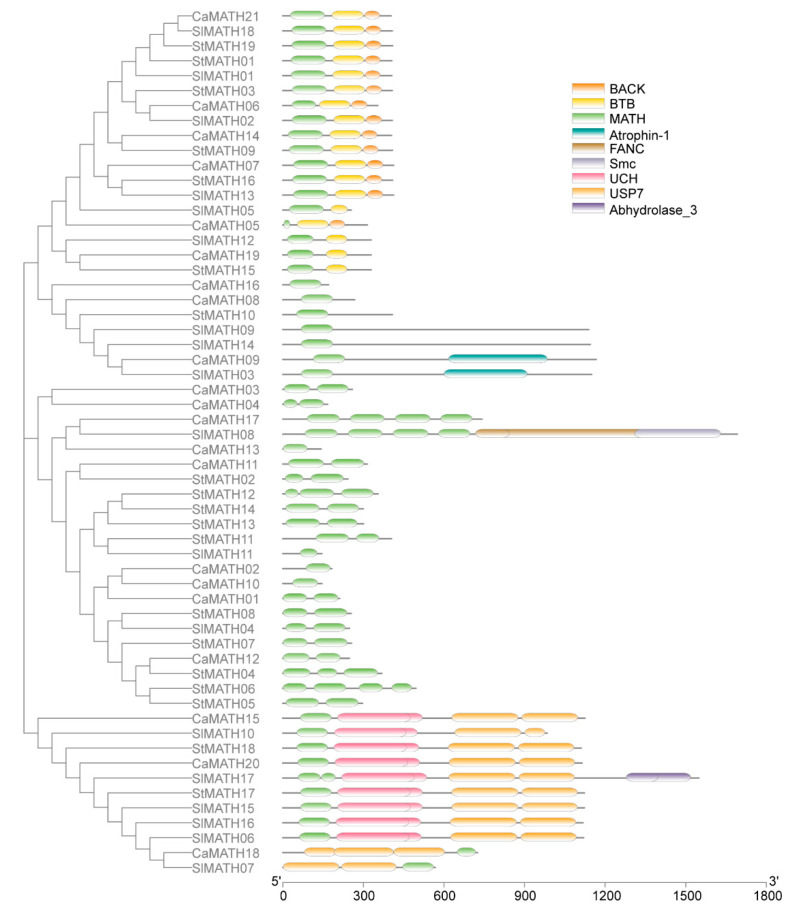
Phylogeny and domain organization of MATH proteins of Solanaceae (tomato, potato, and pepper). (**Left**) Phylogenetic tree of 58 Solanaceae MATH proteins reconstructed using MEGA v11 software. (**Right**) Conserved domains in these Solanaceae MATH proteins indicated using NCBI-CDD search.

We then constructed another phylogenetic tree using the 58 Solanaceae MATH proteins alone to confirm the phylogenetic relationship among the Solanaceae members. This analysis, combined with the domain annotation, clustered Solanaceae MATHs into four groups (Figure 2), consistent with the ML tree of MATH proteins from eight species (Figure 1). In Solanaceae, the MATH-only protein was the largest type and consisted of 29 members classified into two groups; one group contained three SlMATHs, three CaMATHs, and one StMATH, which were single-MATH-domain proteins, and the other group included 10 StMATHs, nine SlMATHs, and three CaMATHs, which were multiple-MATH-domain proteins (Figure 2). The second largest protein family, MATH-BTB, had six members in each of the three Solanaceae plants, all clustered into the same group (Figure 2). In addition, the remaining 11 members, the MATH-USP7-type proteins (six SlMATHs, three CaMATHs, and two StMATHs), were also clustered into the same group (Figure 2). These results, together with the phylogenetic tree of MATH proteins from eight species, suggested a significant expansion of MATH-only proteins in Solanaceae, similar to other plant species, except for the two green algae. 

**Figure 3:**
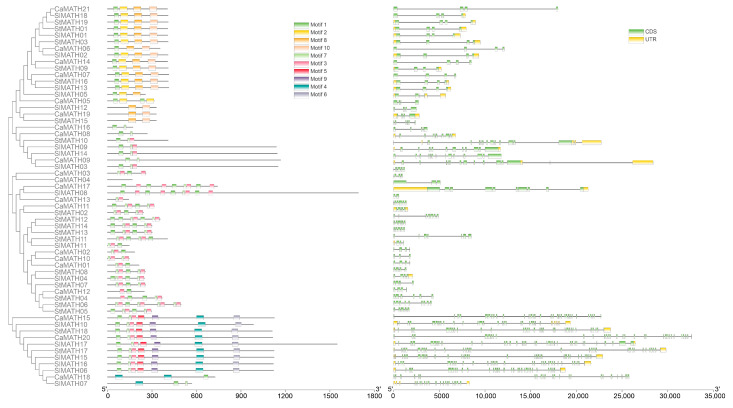
Protein motif and gene structure analysis of Solanaceae MATH members. (**Left**): Phylogenetic tree of 58 Solanaceae MATH members. (**Middle**): Motif distribution in the MATH proteins detected using MEME suit. The different motifs are shown as blocks in different colors. (**Right**): Exon–intron organization of MATH genes, according to the GSDS website. The filled boxes and gray lines represent exons and introns, respectively.

Furthermore, we analyzed the conserved motifs of each protein using the multiple EM for motif elicitation (MEME v5.5.2) tool. The arrangement of ten motifs was consistent with the ML-based phylogenetic results of the 58 Solanaceae MATH proteins (Figure 3). Motif 1 was present in most of the Solanaceae MATH members, while motifs 3 and 7 were present primarily in MATH members of three groups excluding the MATH-BTB group. Motifs 2, 8, and 10 were found exclusively in the MATH-BTB group, whereas motifs 4–6 and 9 were distributed explicitly in the MATH-USP7 group (Figure 3). Then, we explored the exon–intron organization of the Solanaceae MATH genes using the gene structure display server (GSDS). The analysis showed that exons in these genes varied from three to thirty-two (Figure 3). All the MATH-BTB genes showed similar gene structure and exon number, suggesting conservation among these genes in Solanaceae. Maximum introns were found in the MATH-USP7 subgroup. These observations suggested diversity among the MATH gene family members in Solanaceae.

### 2.3. Gene Duplication and Synteny Analysis of the MATH Gene Family in Solanaceae

**Figure 4:**
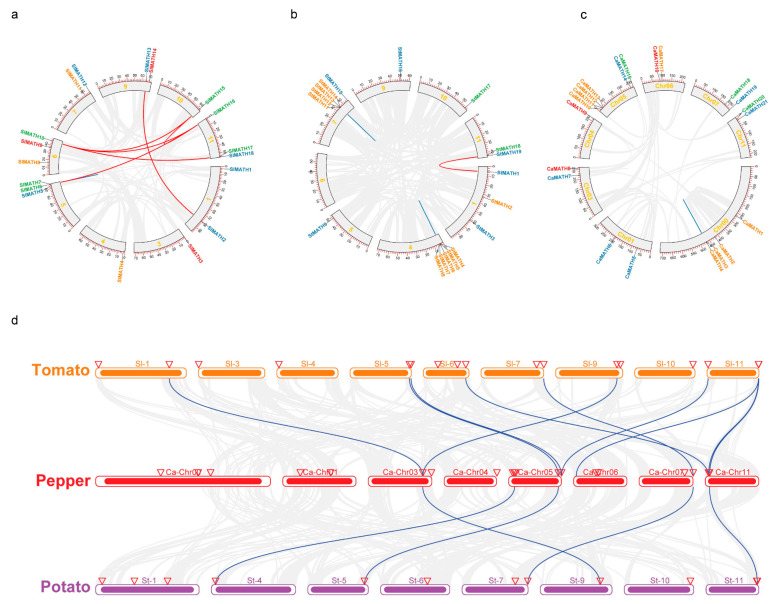
Chromosome mapping, gene duplication, and synteny analyses of MATH genes in Solanaceae. (**a**–**c**) Chromosomal localization and interchromosomal relations of MATH genes in tomato, potato, and pepper. MATH genes were mapped to the chromosomes of tomato (**a**), potato (**b**), and pepper (**c**) and numbered according to their position on the chromosomes. The text colors indicate different types of MATH genes: red, single-MATH; organe, multiple-MATH; blue, MATH-BTB; green, MATH-USP7. Segmental duplications are shown in red lines, and tandem duplications are in blue lines. (**d**) Synteny and collinearity analyses of MATH genes across three Solanaceae species. The gray lines in the background indicate the putative orthologous genes, and the blue lines represent the syntenic MATH gene pairs.

To determine the chromosomal distribution of the Solanaceae MATH genes, we detected the chromosome localization of each gene based on the annotation of each genome. All 58 Solanaceae MATH genes were localized on 7–9 chromosomes of each genome (Figure 4). In tomato, *SlMATH* genes were mainly distributed on chr5, chr6, and chr11, each containing three members; chr1, chr7, and chr9 had two genes each, whereas chr3 and chr5 had another two genes each (Figure 4a). In the potato, *StMATH* genes were more concentrated on five chromosomes, chr1, chr4, chr7, and chr11, which contained 15 members. Here, chr5, chr6, chr9, and chr10 had only one gene each (Figure 4b). Similarly, the five chromosomes of pepper, chr01, chr03, chr06, chr07, and chr11, contained two *CaMATH* each; chr00 and chr05 contained eight genes each; and chr04 had only one gene (Figure 4c).

We performed the intraspecific collinearity analysis of three Solanaceae species using the MCScanX program to further investigate the relationships among Solanaceae MATH genes. The results revealed four tandem duplication events of *MATH* genes in Solanaceae (Figure 4 and Table S3), including one (*SlMATH06/07*) in tomato (Figure 4a), two (*StMATH04*/05/06/0*7*/0*8* and *StMATH11*/*12*/*13*/*14*) in potato (Figure 4b), and one (*CaMATH03*/*CaMATH04*) in pepper (Figure 4c). Among these tandem duplicated genes, the *SlMATH05/06* gene pair belonged to the MATH-USP7 group, while the other nine tandem-duplicated genes belonged to the MATH-only group (Figure 2). In addition, six segmental duplicated gene pairs were found in the tomato, but only one was found in the potato, and none in the pepper (Figure 4 and Table S3). Of those, four pairs of *SlMTAH* genes (*SlMATH15*/*SlMATH16*, *SlMATH15*/*SlMATH10*, *SlMATH16/SlMATH6*, and *SlMATH17/ SlMATH10*) from tomato (Figure 4a) belonged to the MATH-USP7 subgroup, while two pairs (*StMATH01/StMATH19* and *SlMATH02/SlMATH13*) (Figure 4a,b) belonged to the MATH-BTB subgroup (Figure 2). These results suggested that segmental duplications rather than tandem duplications prompted the expansion of the MATH gene family in tomato, while tandem duplications predominantly promoted gene expansion in potato. 

To further examine the origin and evolutionary history of Solanaceae MATH genes, we performed interspecific collinearity analyses to determine the synteny and collinearity degree of MATH genes across Solanaceae. In total, 28 orthologous MATH gene pairs were found in Solanaceae genomes, including 13 blocks between tomato and potato, 10 between tomato and pepper, and five between potato and pepper (Figure 4d). Interestingly, *CaMATH* and *StMATH* from each of the four orthologous gene pairs (*CaMATH07*/*StMATH16*, *CaMATH14*/*StMATH09*, *CaMATH19*/*StMATH15*, and *CaMATH21*/*StMATH19*) exhibited collinearity with the same *SlMATH* of the four genes (*SlMATH13*/*SlMATH18*/*SlMATH12*/*SlMATH18*), suggesting that MATH members of these four collinear gene pairs across the three Solanaceae species might have been derived from common ancestors of these plants.

### 2.4. Cis-Regulatory Elements in MATH Promoters

**Figure 5:**
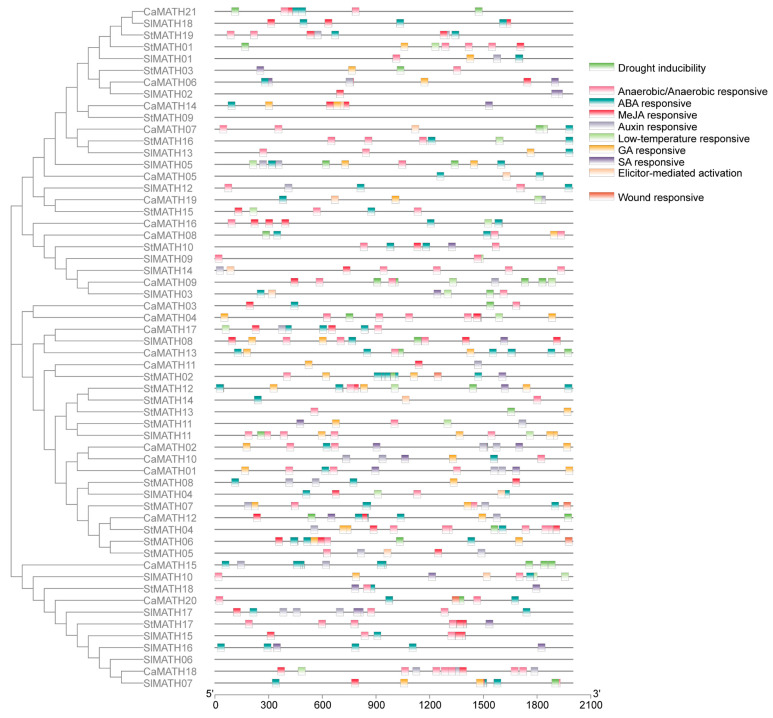
Regulatory elements in the promoters of Solanaceae MATH genes. (**Left**) Phylogenetic tree of 58 Solanaceae members. (**Right**) The *cis*-regulatory elements in the MATH promoters, predicted by the PlantCARE database. The different CREs in the promoter regions are shown as blocks in different colors.

MATH genes play important roles in plant development and defense responses [8]. Therefore, to explore the potential functions of Solanaceae MATH genes involved in these processes, we predicted the *cis*-regulatory elements (CREs) in the 2 kb upstream promoter sequences of these genes using the PlantCARE database. This analysis showed ten distinct elements classified as phytohormone-responsive (five elements) and stress-responsive (five elements), relevant to development and stress response. A total of 491 potential CREs were identified across the promoter regions of the 58 Solanaceae MATH genes (Figure 5).

Among these CREs, 120 putative ABA-responsive elements (ABREs) were found in the promoters of 45 MATH genes, suggesting their roles in responding to ABA-involved stress, such as salt and drought stresses (Figure 5). Consistent with this observation, 31 putative drought-responsive elements (MYB binding sites and MBSs) were found in the promoters of 21 MATH genes. Furthermore, 20 MATH gene promoters contained 45 GARE (gibberellin-responsive) motifs, and 46 contained 120 putative anaerobic- or anoxia-responsive elements (AREs) (Figure 5). Notably, 104 MeJA-responsive elements (TGACG or CGTCA motifs) were found in the promoters of 34 MATH genes, and 26 SA-responsive elements (TCA elements) were found in those of 19 members; only 10 auxin-responsive elements (AuxRR-core motifs) were found in seven gene promoters (Figure 5). In addition, more than 60 stress-related putative responsive elements, including 21 low-temperature-responsive elements (LTREs), 30 defense- and stress-responsive elements (TC-rich repeats), 10 elicitor-mediated activation elements (AT-rice sequence), and four wound-responsive elements (WUN-motif), were identified in the promoters of 42 MATH genes (Figure 5). These results suggested that Solanaceae MATH potentially functions in responding to stress-related phytohormone signaling and biotic and abiotic stresses.

### 2.5. Tissue-Specific Expression Patterns of Solanaceae MATH Genes

**Figure 6:**
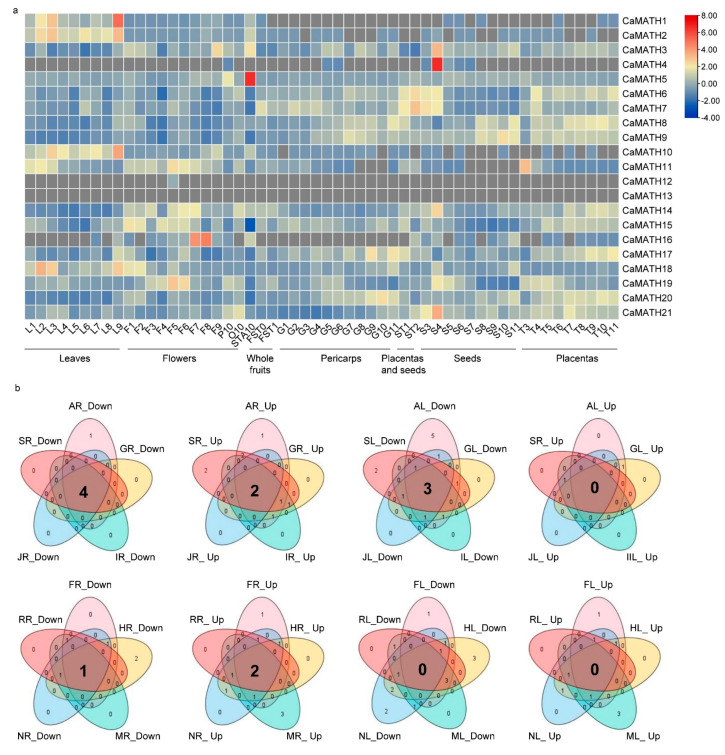
Tissue-specific expression of *MATH* genes in pepper. (**a**) Heatmap showing the expression of *CaMATH* genes in 54 samples from different tissues or organs of pepper during successive developmental stages, including leaves (L1−L9), flower-related organs [flowers (L1−L9), petal (P10), ovary (O10), anther (STA10)], and 33 fruit-related tissues [whole fruits (FST0−FST1), pericarps (G1−G11), placenta and seed tissues (ST1−ST2), placenta (T3−T11), and seeds (S3−S11)]. The heatmap was drawn using FPKM values from previously reported RNA-seq data in the Pepperhub database using the TBtools v1.108 software. (**b**) Venn diagrams based on *CaMATH* expression in response to phytohormones and various stresses. A: ABA-treated; S: SA-treated; J: JA-treated; I: IAA-treated; G: GA-treated; F: freezing-treated; R: H_2_O_2_-treated; N: NaCl-treated; M: mannitol-treated; H: heat-treated; Up: upregulated genes; Down: downregulated genes. L: leaves; R: roots. The two-letter code lists the treatment first, then the tissue. The numbers given in the Venn diagram represent the numbers of upregulated/downregulated genes.

To investigate the potential functions of Solanaceae MATH genes in plant development, we explored their expression profiles across diverse organs during different developmental stages using previously published transcriptome data of tomato (CRA001723 and CRA001712) [30], potato (SRA049915, GSE33507, SRA050797, and SRA048144) [14], and pepper (CRA001412) [31]. Based on the pepper tissue-specific expression data in the transcriptome module of the PepperHub database containing more than 50 samples, a heatmap was generated to show the expression profiles of 21 *CaMATH* genes across organs during development (Figure 6a). *CaMATH06* displayed higher expression levels than other genes in most samples, especially in the petal (P10), ovary (O10), whole fruit (FST0–FST1), pericarp (G1–G10), placenta and seed combination (ST1–ST2), placenta (T3–T11), and seed (S3–S4) (Figure 6a). However, the expression levels of *CaMATH06* were lower in the seeds at the middle and later stages of development (S5–S11) than in the S3–S4 and ST1–ST2 samples (Figure 6a). These observations implied important roles of *CaMATH06* in regulating fruit development, especially the differentiation of the placenta and seed. Interestingly, *CaMATH11* exhibited higher transcript levels in the early stages of the leaf (L1–L3) than in the middle and later stages (L4–L9). On the other hand, *CaMATH20* expression levels were higher in the middle and later stages of the pericarp (G5–G11) and placenta (T5–T11) than in early stages (Figure 6a). These results suggested a negative role for *CaMATH11* but a positive role for *CaAMTH20* in modulating the senescence and maturity of pepper tissues. In addition, *CaMATH01*, *CaMATH02*, and *CaMATH10* showed higher expression in the leaf than in other tissues, and *CaMATH05* showed higher expression in the petal (P10) and anther (STA10) than in other tissues (Figure 6a), implying their potential roles in regulating the development of these specific tissues.

Tomato transcriptome data were collected from 20 different tissues and stages, including roots, stems, and leaves at bud, flowering, and breaker stages; bud and flower samples from seedlings at bud and flowering stages; and pericarp at nine different time points of fruit development. Gene expression analysis revealed that 14 of 18 *SlMATHs* were highly expressed in most tissues. In contrast, the expression levels of the other four *SlMATHs* (*SlMATH04*/07/10/11) were either low or negligible, except for *SlAMTH10* and *SlAMTH11* in F30 (the leaf sample from the bud stage at 30 days post-germination) (Figure S1a), suggesting considerable roles of *SlMATHs* during development in the tomato. Furthermore, the heatmap drawn based on the transcriptome data of 13 selected potato tissues showed poor or no expression of 11 *StMATHs* in these tissues. However, the remaining eight genes were highly expressed in most tissues; *StMATH16* showed the highest expression in all 13 tissues (Figure S1b), implying its important roles in vegetative and reproductive development in the potato.

### 2.6. Expression of MATH Genes in Solanaceae Plants in Response to Phytohormone Treatment and Various Stresses

Given the vast number of phytohormone- and stress-response elements found in the promoter regions of Solanaceae *MATH* genes, we analyzed the expression patterns of *StMATH* and *CaMATH* genes after stress exposure and phytohormone treatment using previously published datasets of potato [14] and pepper [31], as we did not find suitable stress-related transcriptome data in tomato databases. The pepper stress- and phytohormone-related transcriptome data in the PepperHub database included leaf and root samples exposed to various phytohormones (ABA, GA, IAA, JA, and SA) and stresses (cold, H_2_O_2_, heat, mannitol, and NaCl). Analysis of this data showed that 16 *CaMATH* genes were regulated by phytohormones or stresses (Figure 6b and Table S4). In leaves, most *CaMATH* genes were repressed by more than one phytohormone or stress treatment, with only four CaMATH genes induced upon one or two treatments (Figure 6b and Table S4). Unlike in leaves, roots showed upregulation of 12 *CaMATH* genes and downregulation of eight genes after at least one phytohormone or stress treatment (Figure 6b and Table S4). Interestingly, in roots, *CaMATH08* and *CaMATH17* were upregulated while *CaMATH02* was downregulated under all ten treatments, while *CaMATH01*, *CaMATH05*, and *CaMATH10* were downregulated under all phytohormone treatments (Figure 6b and Table S4). In leaves, *CaMATH01*, *CaMATH05*, and *CaMATH17* were downregulated under all phytohormone treatments. Furthermore, *CaMATH01* and *CaMATH05* expression patterns in the leaf were similar to those in the root, whereas *CaMATH17* displayed an opposite expression pattern (Figure 6a). These results suggested distinct roles for *CaMATH* genes in the root and leaf of pepper in response to phytohormone treatment and stresses.

The potato transcriptome dataset included data after various stress and phytohormone treatments, including salt, mannitol, heat, *P*. *infestans*, IAA, GA, and ABA. Analysis of this dataset showed that *StMATH10*, *StMATH17*, and *StMATH19* were induced by both salt and mannitol treatments, and *StMATH01* and *StMATH11* were upregulated only during salt treatment. However, *StMATH16* decreased during both treatments, while *StMATH03* and *StMATH9* were reduced only by mannitol treatment (Figure S1c). Heat treatment decreased the expression levels of *StMATH01*, *StMATH02*, *StMATH04*, and *StMATH16*, and *P*. *infestans* infection downregulated *StMATH10*, *StMATH11*, *StMATH15*, and *StMATH17* (Figure S1c). In addition, ABA treatment induced the expression levels of *StMATH08*, *StMATH10*, *StMATH11*, and *StMATH15*, while GA_3_ treatment increased the expression levels of *StMATH02*, *StMATH11*, and *StMATH15*. However, IAA treatment intensely suppressed only *StMATH11* (Figure S1c). These results suggested that *StMATH* genes may play important roles in regulating stress response in potato.

### 2.7. Expression of Pepper MATH Genes during Development and in Response to Flooding Treatment

**Figure 7:**
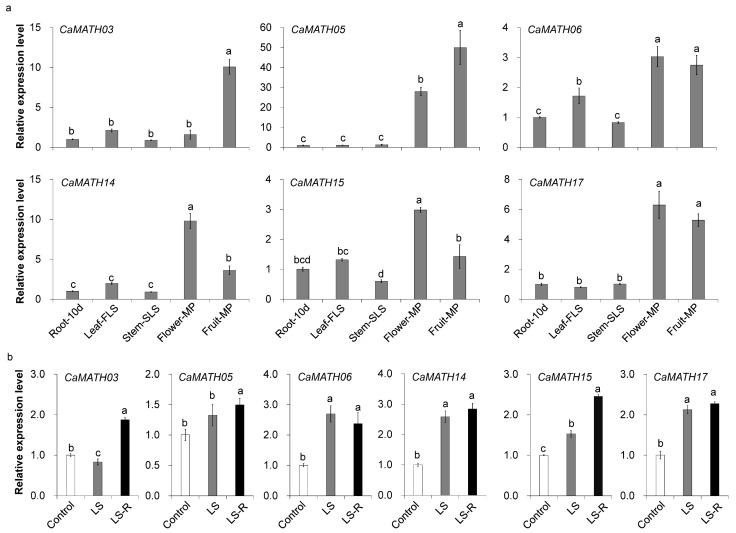
Expression pattern of *CaMATH* genes in pepper during development and after light flooding treatment. (**a**,**b**) Quantitative reverse transcription PCR (qRT-PCR) analysis of six CaMATH genes in different tissues (root, leaf, stem, flower, and fruit) (**a**) and after light flooding treatment (**b**). The *CaUBI-3* gene was used as the internal reference to normalize the gene transcript levels. Each data point represents the average of three biological repeats. Lowercase letters indicate significant differences between treatment groups based on one-way ANOVA (*p* < 0.05). 10d: 10 days after germination; FLS: four-leaf stage; SLS: six-leaf stage; MP: mature period; LS: light flooding treatment; LS-R: recovery after LS treatment.

To elucidate the role of *CaMATH* during pepper development, we analyzed the expression levels of six *CaMATH* genes in different tissues and organs, including root, leaf, stem, flower, and fruit. Quantitative reverse transcription PCR (qRT-PCR) showed that *CaMATH14* and *CaMATH15* were highly expressed in flowers, and *CaMATH03* was preferentially expressed in mature fruit. *CaMATH05*, *CaMATH06*, and *CaMATH17* exhibited higher expression levels in flower and mature fruit than in other tissues (Figure 7a), consistent with the PepperHub RNA-seq data (Figure 6a).

We also tested expression levels of these selected *CaMATH* genes in pepper seedlings under light flooding (LS) treatment and during recovery after LS treatment, as various anaerobic- or anoxia-responsive elements were found in the promoters of most *CaMATH* genes (Figure 5). The qRT-PCR analysis showed that three of these selected *CaMATH* genes, *CaMATH06*, *CaMATH14*, and *CaMATH15*, were significantly induced at 48 h upon LS treatment compared to the control. In addition, the transcript levels of all six genes were significantly high at recovery (24 h after LS treatment), suggesting the potential role of *CaMATHs* in the submergence and re-submergence response. Taken together, the qRT-PCR results confirmed that *CaMATH* genes play important roles during development and in regulating stress response in pepper.

## 3. Discussion

MATH proteins have been found to play vital roles in regulating plant development, phytohormone signaling, immunity, and abiotic stresses by acting as molecular adaptors or ubiquitin E3 ligases [2,3,5,8]. Researchers have identified MATH genes in several plant species, such as Arabidopsis [9], *Brassica rapa* [7], rice [10], and maize [11]. However, the MATH gene family has not been comprehensively identified in Solanaceae, an economically important crop family. In this study, we identified and systematically analyzed MATH genes in three representative Solanaceae species, including tomato, potato, and pepper. We also investigated the evolutionary relationship and expression profiles of Solanaceae MATH genes. These results lay a foundation for further study of the functions of these genes in plant development and stress responses.

### 3.1. Classification and Conserved Nature of MATH Gene Family in Solanaceae

Combining BLASTP searches with HMMER model analysis, we identified 161 MATH genes in eight selected plant genomes, among which 58 were found in Solanaceae genomes. The occurrence of the MATH gene was detected in all eight species, especially in two green algae (Table 1), suggesting that the MATH gene family is conserved across plants. We identified 18 MATH genes in tomato, 19 in potato, and 21 in pepper, while the genome sizes of potato (900 Mb) [14] and tomato (844 Mb) [15] are smaller than that of pepper (3260 Mb) [16,17] (Table 1). Thus, our study suggests a similar number of MATH members in Solanaceae, irrespective of genome size. Studies have reported over 70 members of the MATH family in some Brassicaceae and Poaceae plants, including *Brassica rapa*, Arabidopsis, and rice [7,8,9,10]. Compared to these Brassicaceae and Poaceae plants, the fewer MATH members in tomato, potato, and pepper suggest a contraction of the MATH gene family in Solanaceae plants. 

In plants, MATH-only proteins include those with single-MATH domain and multiple-MATH tandem repeats [7,8,10]. Consistent with these earlier reports, our study also detected MATH-only proteins with single- or multiple-MATH domains in six terrestrial plant species. However, the MATH-only proteins from the two green algae were identified as single-MATH-domain proteins. This domain annotation result suggests that the proteins with multiple-MATH domains are specific to terrestrial plant species. As a conserved protein family in eukaryotes, MATH proteins, excluding MATH-only proteins, are often associated with a set of functional protein domains, such as BTB and USP7 [2,8]. Based on the associated domains, MATH proteins have been mainly classified into seven families. However, the MATH-RluA family; TRIM37 family; and Meprin family, which was found in *Alveolata* or vertebrates [5], have not been identified in the selected plants. Zhao et al. (2013) grouped MATH proteins into six clusters in *Brassica rapa* [7]. Group I included MATH-domain-only proteins with multiple-MATH domains in tandem, group II included MATH-BTB proteins, group III included MATH-domain-only proteins with four-MATH domains in tandem, group IV included single-MATH-domain proteins and some proteins with PEARL-4 domain, group V included single-MATH-domain proteins, and group VI included MATH-USP7 proteins [7]. A recent study revealed that MATH proteins in Arabidopsis and rice are clustered into four groups: MATH-only proteins, MATH-BPM proteins, MATH-UBP proteins, and MATH-PEARLI-4 proteins [8].

The present study grouped the MATH proteins from eight plant species into six subclades belonging to four groups: single-MATH-domain proteins, MATH-USP7 proteins, MATH-BTB proteins, and multiple-MATH-domain proteins (Figure 1). In the single-MATH-domain protein group, one subclade had Arabidopsis MATH proteins and some Arabidopsis MATH-PEARL4 proteins [8]. However, the MATH-PEARL4-type protein has not been reported in other plant species (Figure 1). In addition, ten four-MATH-domain proteins from six plant species were clustered into an individual subclade in the multiple-MATH-domain protein group (Figure 1). Furthermore, the specific phylogenetic tree of 58 Solanaceae MATH proteins demonstrated that Solanaceae MATH proteins were also classified into four groups without the MATH-PEARL4 and four-MATH-domain subclades (Figure 2). Thus, in the present study, the phylogeny of MATH members in three Solanaceae species and other plant species was more consistent with the classification of MATH proteins in *Brassica rapa* [7].

### 3.2. Gene Duplications Contributed to MATH Gene Expansion in Solanaceae

The 58 MATH genes from three Solanaceae species were mapped onto 7–9 chromosomes within each genome; however, there were two gene clusters in the potato genome and one gene cluster in the pepper genome, suggesting that MATH genes were unevenly distributed across the Solanaceae chromosomes (Figure 3). Gene duplication is a major factor responsible for the expansion of the gene family, and environmental and biological factors in the host organism regulate this. The analysis of MATH gene duplication in Solanaceae genomes revealed four tandem duplication events and six gene pairs with segmental duplications in Solanaceae (Figure 3), suggesting that both segmental duplication and tandem duplication are responsible for the expansion in Solanaceae MATH genes. Among these genes in duplication events, nine *StMATH* genes from two tandem-duplicated events were clustered into the multiple-MATH-domain group, and five *SlMATH* genes from four segmental-duplication gene pairs belonged to the MATH-USP7 group. Overall, our results indicated that segmental duplications rather than tandem duplication led to the expansion of the MATH-USP7 gene subfamily in tomato. In contrast, tandem duplication resulted in the expansion of the multiple-MATH-domain gene subfamily in potato.

Proteins with MATH and BTB/POZ domains are widely found in eukaryotes. Previous studies identified six MATH-BTB domain proteins in Arabidopsis [8,10] and ten in *B*. *rapa* [7]. Several other studies have shown that the MATH-BTB gene was the largest subfamily in grasses but not in Arabidopsis, banana, *B*. *rapa*, and other lower plants, suggesting a monocot-specific expansion of MATH-BTB genes [7,10,12,32]. In this study, we found six MATH-BTB genes in each of the three selected Solanaceae with different genome sizes; these genes were clustered into one group without any indication of gene expansion (Table 1 and Figure 1). However, one segmental-duplicated gene pair was found in tomato and potato. Notably, the similarities in gene length, exon–intron structure, and protein motif organization supported the highly conserved nature of MATH-BTB genes in Solanaceae (Figure 3). Thus, our results indicate that MATH-BTB genes in Solanaceae remain evolutionarily conserved; however, their associated functions are unclear.

### 3.3. MATH Predicted Functions and Gene Expression

Several studies have shown that plant MATH proteins play versatile roles in modulating developmental processes [8]. There were two Arabidopsis MATH-USP7 genes (UBP12, AT5G06600 and UBP13, AT3G11910) in group II, with two, three, and two MATH-USP7 genes in pepper, tomato, and potato, respectively, suggesting high conservation among those plants. UBP12 and UBP13 have been reported to function as regulators in restricting protease activity of their distinct target proteins through deubiquitylation during leaf development, root development, and flowering time [23,24,25], implying that their homologous gens in Solanaceae displayed a similar function, beneficial topic for further research.

MATH proteins also function as critical regulators of biotic and abiotic stress responses in plants [8]. MATH-only proteins AtTRAF1a and AtTRAF1b (also named AtMUSE14 and AtMUSE13) regulate plant autoimmunity and autophagy-mediated nutrient deprivation by acting as molecular adaptors associated with E3 ubiquitin ligases [18,19,20,21]. Expression profiling generally provides valuable clues for inferring the functions of genes. The present study analyzed Solanaceae MATH gene expression using publicly available RNA-seq data, with the result that more than ten *CaMATH* genes were upregulated after exposure to at least one stress factor or treatment with one phytohormone (Figure 6b), and several *StMATH* genes were found to be induced or reduced by different stress and phytohormone treatments (Figure S1c). In addition, our qRT-PCR experiment illustrated that six *CaMATH* genes were highly induced after LS treatment, suggesting crucial roles for Solanaceae MATH genes upon stress treatment, although their function in stress response remains to be further elucidated.

## 4. Materials and Methods

### 4.1. Identification of MATH Genes

We used known Arabidopsis MATH domain genes [9] to query using the BLASTP method against the sequenced representative genomes. The genomes of two green algae (*Ostreococcus lucimarinus* and *Chlamydomonas reinhardtii*); one moss (*Physcomitrella patens*); spikemoss (*Selaginella moellendorffii*); rice (*Oryza sativa*); and three Solanaceae species, including tomato (*Solanum lycopersicum*), potato (*Solanum tuberosum*), and pepper (*Capsicum annuum*), were downloaded from the Ensembl Plants public database (http://plants.ensembl.org/index.html, 6 June 2020) [33]. The HMM profile (Pfam entry PF00917) of the MATH domain downloaded from the Pfam database [34] was used as a seed to identify MATH candidate proteins from the three Solanaceae species with HMMER 3.0 software [35], with an E-value cutoff of 10^−5^ [36]. Finally, each non-redundant candidate protein was verified based on the presence of a complete MATH domain. We also identified other conserved functional domains in the sequences based on the SMART and CDD databases, as described previously [37,38].

### 4.2. Protein Property Predictions

The molecular weight (Mw) and theoretical isoelectric point (pI) of 58 Solanaceae MATH proteins were predicted using the ‘Prosite’ online tool in Expasy (https://prosite.expasy.org, 12 December 2020) [39]. The subcellular localization of these proteins was predicted based on the online software Plant-mPLoc v2.0 (http://www.csbio.sjtu.edu.cn/bioinf/plant, 1 February 2021) [40]. 

### 4.3. Phylogenetic Analysis

MATH domain sequences of 129 MATH members from eight plant species and 58 Solanaceae MATH members identified in this study were used to construct the unrooted phylogenetic trees. First, the complete protein sequences of MATH members were aligned using ClustalX 2.0 [41] based on the BLOSUM 30 protein weight matrix and default gap extension penalty. Then, the unrooted phylogenetic trees were constructed with MEGA v11 software [42], using the maximum likelihood method (ML) based on the Jones–Taylor–Thornton (JTT) matrix-based evolutionary model with pairwise deletions and 1000 bootstraps. Finally, the phylogenetic trees (161 MATH proteins from eight plant species and 58 MATH proteins from three Solanaceae species) were optimized using FigTree v1.4.4 software.

### 4.4. Duplication Events and Synteny Analysis

The complete genome sequences and annotation files of Arabidopsis, rice, and the three Solanaceae species obtained from the Ensembl Plants database were used to perform synteny analysis with MCScanX software and identify intragenomic (evidence of whole-genome duplications) and intergenomic (between related species) syntenic blocks of Solanaceae *MATH* genes. The segmental duplication in all MATH genes was determined by expanding the analysis to the ten genes flanking each *MATH* gene [43]. Finally, the duplication events and synteny analysis were visualized and illustrated using TBtools v1.108 software [44].

### 4.5. Analysis of Protein Motif and Domain Combinations, Gene Exon–Intron Architecture, and Promoter Cis-Elements in Solanaceae MATH Members

The conserved motifs and functional domains of 58 Solanaceae MATH proteins were identified with MEME v5.5.2 online software (https://meme-suite.org/meme/tools/meme, 27 July 2020) [45] and protein databases SMART and CDD. The exon–intron organization of these Solanaceae MATH genes was predicted using the Gene Structure Display Server (GSDS2.0). Finally, the protein motifs and domains and the gene exon–intron organization were visualized and illustrated using TBtools [44] combined with the unrooted phylogenetic tree of these MATH proteins. In addition, the 2000 bp upstream region of the coding region of each of the 58 Solanaceae *MATH* members was defined as the promoters and submitted to the PlantCARE database (http://bioinformatics.psb.ugent.be/webtools/plantcare/html, 21 January 2021) to identify the putative *cis*-regulatory elements [46]. The *cis*-regulatory elements and the phylogenetic tree were also visualized using TBtools.

### 4.6. Analysis of Gene Expression during Different Developmental Stages and under Different Stress and Phytohormone Treatments

The tissue-specific and stress/phytohormone treatment-dependent expression patterns of *MATH* genes in Solanaceae were explored by reanalyzing the published transcriptome (RNA-seq) or microarray datasets for tomato [30], potato [14], and pepper [31]. The TPM (transcripts per million) values of all the transcripts were collected from the tomato transcriptome dataset (20 tissues) of distinct tissues during different development stages [30], including roots, stems, and leaves at three major growth stages (bud stage, flowering stage, and breaker stage); buds and flowers from seedlings at bud and flowering stage; and pericarps at nine different time points of fruit development. The FPKM values of all potato transcripts were obtained from the public dataset of 13 tissues [14], including roots, shoots, stolons, leaves, petioles, flowers, petals, stamens, sepals, carpels, immature fruits, and tubers. This dataset was also used to obtain the expression patterns under various stress and phytohormone treatments, including 24 h after salt, mannitol, heat, *P*. *infestans*, IAA, GA, and ABA treatments. The FPKM values for pepper were obtained from the transcriptome dataset in the PepperHub database [31] with 188 samples of different organs/tissues during consecutive development stages or different stress and phytohormone treatments. In this study, all tissue samples and treatment samples of leaves were reanalyzed to reveal expression patterns of *CaMATH* genes. The heatmap and Venn diagrams of expression profiles of 58 Solanaceae *MATH* genes were visualized and illustrated using TBtools v1.108 software [44].

### 4.7. RNA Extraction and Quantitative RT-PCR

Seedlings of the pepper cultivar Capsicum 6421 were grown in a greenhouse under 25/23 °C (day/night) and a photoperiod of 16 h light/8 h darkness. RNA was extracted from the pepper roots (ten days after germination and four-leaf stage), leaves (four-leaf stage), stem (six-leaf stage), flowers (mature period), and fruits (mature period) to determine gene expression patterns [47]. For a light flooding treatment, four-week-old pepper seedlings were submerged 10 cm under the water surface in normal light, and then the leaf samples were collected 48 h after submergence and 24 h after de-submergence [48,49]. The qRT-PCR analysis was performed as previously described [49,50], using primers obtained from the MRPrimerW2 database [51] (Table S5) and *CaUBI*-*3* as the internal reference.

## 5. Conclusions

Here, we characterized 58 MATH genes in three representative Solanaceae species and divided them into four groups based on phylogenetic data, domain organization, motif composition, and gene structure. Intra-genome synteny analysis indicated that both segmental and tandem duplication played important roles during MATH gene expansion in tomato and potato. Our comprehensive analysis also indicated the role of several Solanaceae MATH genes in regulating vegetative growth and environmental adaptation. Thus, the study provides insights into the MATH genes and lays a foundation for further functional characterization of these genes in Solanaceae.

## Figures and Tables

**Table 1:** Comprehensive MATH genes identified in eight selected plants.

Plant Species	Clade	Genome Size (Mb)	Total	One MATH	Two MATH	Three MATH	Four MATH	MATH-BTB	MATH-USP7
*Ostreococcus lucimarinus*	Chlorophytes	12.56	3	1	-	-	-	1	1
*Chlamydomonas reinhardtii*	Chlorophytes	120	8	3	-	-	-	4	1
*Physcomitrium patens*	Bryophytes	472	18	6	1	-	4	4	3
*Selaginella moellendorffii*	Lycophytes	100	7	1	-	-	1	3	2
*Arabidopsis thaliana*	Eudicots	135	67	32	25	-	2	6	2
*Solanum lycopersicum*	Eudicots	844	18	5	1	-	1	6	5
*Solanum tuberosum*	Eudicots	900	19	1	7	2	1	6	2
*Capsicum annuum-Zunla*	Eudicots	3260	21	6	5	-	1	6	3
Total			161	55	39	2	10	36	19

## Data Availability

The data that support the findings of this study are available from the corresponding author upon reasonable request.

## Supplementary Materials

The supporting information can be downloaded at: https://www.mdpi.com/article/10.3390/ijms24108782/s1.
