# Supplementary material for: Evolution and Expression of the Meprin and TRAF Homology Domain-Containing Gene Family in Solanaceae
Source: Int J Mol Sci. 2023 May 15;24(10):8782. doi: 10.3390/ijms24108782 (PMC10218331; doi:10.3390/ijms24108782)
Supplement: Supplementary file 1 [file ijms-24-08782-s001.zip › Figure S1. Expression patterns ofof SlMATH and StMATH genes.pdf]

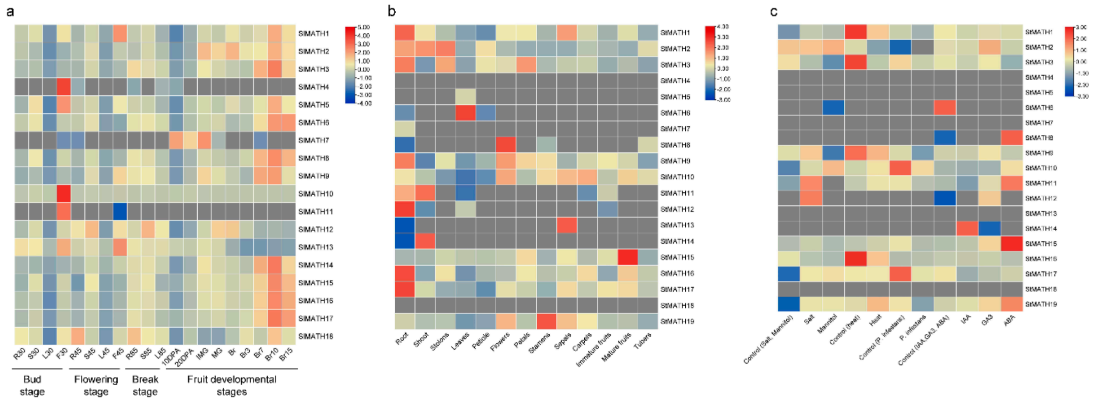

Figure S1. Expression patterns of *SlMATH* and *StMATH* genes during different developmental stages and in response to stress exposure and phytohormone treatment. (**a**) Tissue-specific expression patterns of MATH genes in tomato. Tomato samples: Leaves (L30/L45/L85), roots (R30/R45/R85), stems (S30/S45/S85), bud (F30), and flower (F45) at bud stage [30 days post-germination (DPG)], flowering stage (45DPG), and break stage (85DPG); nine pericarp samples from different fruit developmental stages [10 days post-anthesis (DPA), 20 DPA, immature green (IMG), mature green (MG), breaker (Br), three days after Br stage (Br3), Br7, Br10, and Br15]. (**b**) Tissue-specific expression patterns of MATH genes in potato. Potato samples: 11 samples from distinct organs or tissues, including root, shoot, stolons, leaves, petiole, flowers, petals, stamens, sepals, carpels, immature fruits, mature fruits, and tubers. (**c**) Expression profile of *StMATH* genes in potato under various stresses, such as salt, mannitol, and heat treatment and *P. infestans* infection and after treatment with phytohormones, such as IAA, GA3, and ABA. Heatmaps were drawn using TPM (tomato) or FPKM (potato and pepper) values obtained from previously reported RNA-seq data through the TBtools software.
